# Visualization of hydrocarbon chain length and degree of saturation of fatty acids in mouse livers by combining near-infrared hyperspectral imaging and machine learning

**DOI:** 10.1038/s41598-023-47565-z

**Published:** 2023-11-23

**Authors:** Akino Mori, Masakazu Umezawa, Kyohei Okubo, Tomonori Kamiya, Masao Kamimura, Naoko Ohtani, Kohei Soga

**Affiliations:** 1 Department of Materials Science and Technology, Faculty of Advanced Engineering, Tokyo University of Science, 6-3-1 Niijuku, Katsushika, Tokyo, 125-8585 Japan; 2 Department of Pathophysiology, Graduate School of Medicine, Osaka Metropolitan University, 1-4-3 Asahi-machi, Abeno-ku, Osaka 545-8585 Japan

**Keywords:** Optical imaging, Near-infrared spectroscopy

## Abstract

Fatty acids play various physiological roles owing to their diverse structural characteristics, such as hydrocarbon chain length (HCL) and degree of saturation (DS). Although the distribution of fatty acids in biological tissues is associated with lipid metabolism, in situ imaging tools are still lacking for HCL and DS. Here, we introduce a framework of near-infrared (1000–1400 nm) hyperspectral label-free imaging with machine learning analysis of the fatty acid HCL and DS distribution in the liver at each pixel, in addition to the previously reported total lipid content. The training data of 16 typical fatty acids were obtained by gas chromatography from liver samples of mice fed with various diets. A two-dimensional mapping of these two parameters was successfully performed. Furthermore, the HCL/DS plot exhibited characteristic clustering among the different diet groups. Visualization of fatty acid distribution would provide insights for revealing the pathophysiological conditions of liver diseases and metabolism.

## Introduction

The near-infrared (NIR; 800–2500 nm) region has a longer wavelength than the ultraviolet and visible regions and gives excellent transparency in biological tissues and weak absorption by biomolecules^1^. Absorption attributed to overtones and coupling tones of molecular vibrations in the mid-infrared region occurs in the NIR range. The high transparency of NIR light enables the bioimaging of deep tissues^2^. In contrast, the absorption of various organic substances that appear in the NIR region also facilitates the identification and estimation of the distribution of biomolecules. Owing to these properties, the applications of NIR hyperspectral imaging (HSI) in estimating water content in foods^3^, visualization of chromophores in skin^4^, and diagnostic modalities for otolaryngology^5^ have been reported. However, the absorption spectra of organic substances obtained in the NIR region are complex, with weak and overlapping absorption peaks, which poses a challenge to manual analytical approaches.

Machine learning enables quick and automatic analysis, such as regression for complex data^6^. This approach can be applied to HSI^7^, which provides large-scale data on the optical absorbance of hundreds of wavelength bands for each pixel, that is, two-dimensional position information. The advantage of macroscopic HSI is that, unlike general histopathological diagnosis, it does not require tissue removal^8^. Its application for observation of biological tissues^8^ and cancer diagnosis^9,10^ through machine learning of the numerous amounts of data obtained by HSI, not limited to the NIR wavelength range, has been investigated. Therefore, NIR-HSI can be combined with machine learning to visualize the distribution of organic matter. NIR-HSI with machine learning is applied to food safety and quality inspection^11^ and medical diagnosis, such as the detection of submucosal tumors^12^.

Recently, we reported that NIR-HSI enables imaging of the distribution of lipid concentrations in the liver without labeling or staining^13^. While this study demonstrated the capability of NIR-HSI to map the distribution of whole lipids, it is necessary to visualize lipids with various properties, such as molecular weight, single bonds, and double bonds for each component, and estimate the risk of progression of non-alcoholic fatty liver disease (NAFLD), steatohepatitis (NASH), and NAFLD/NASH-associated liver cancer.

**Figure 1:**
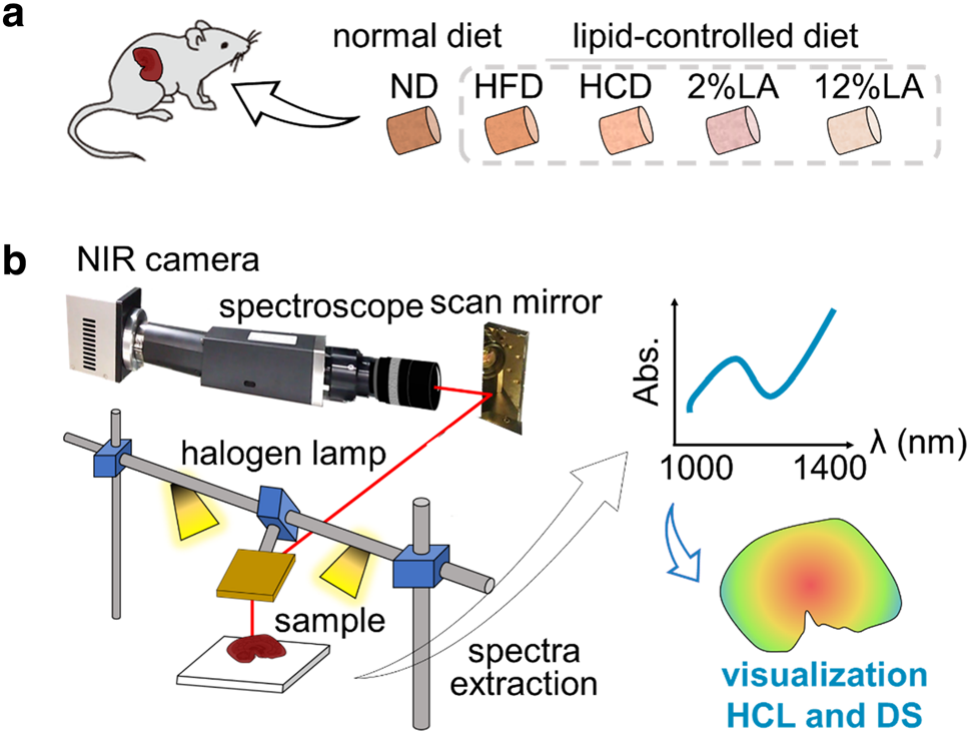
Schematic illustration for NIR-HSI analysis of the liver of mice fed with different diets. (**a**) Specimen: Liver lobes of adult mice with normal and lipid-modified diets (HFD, HCD, and diet with 2% or 12% LA [18:2 (n-6)]) were supplied for the observation of the NIR-HSI and fat component analysis. (**b**) Capturing the NIR-HSI and subsequent analysis: ex vivo liver lobe samples were subjected to NIR-HSI analysis. The light from a specimen is reflected by an angle-variable gold mirror and guided to a spectroscopic camera through a slit. NIR spectral data from 1000 to 1400 nm was obtained for each pixel of the image. The data were analyzed by applying an SVR model for estimating the HCL and DS values for each pixel. The training data for machine learning included the composition of 16 typical fatty acids (Suppl. Table S2), which was provided by GC of samples from each lobe (Suppl. Fig. S1).

Here, we developed a method for label-free imaging of the hydrocarbon chain length (HCL) and degree of saturation (DS) of fatty acids, in addition to the total lipid content, as a first step toward differentiating the distribution of lipids by chemical structure using the NIR-HSI technique coupled with machine learning. We introduced support vector regression (SVR) as a supervised machine learning tool, which is based on structural risk minimization and can solve nonlinear problems with high dimensionality and few samples^14,15^. SVR analysis of NIR-HSI is a novel label-free analysis technique that visualizes the total lipid content in tissues as well as the structural characteristics of fatty acids such as HCL and DS from the NIR reflectance spectra of the tissues (Fig. 1). Beyond the methods used previously^13,16,17^, our method can provide in situ information on HCL and DS of fatty acids in tissues by analyzing NIR hyperspectral images.

## Results

### Determination of the HCL and DS of fatty acids in the liver

First, the fatty acid content in each hepatic lobe was analyzed by GC to obtain the training data for establishing a regression model for the NIR-HSI data to predict the HCL and DS. Liver samples were collected from mice fed with either a normal diet (ND), high-fat diet (HFD), high-cholesterol diet (HCD), or HFD with 2% or 12% linoleic acid (2% LA or 12% LA) (Suppl. Table S1). Each fatty acid was quantified relative to the total fatty acid content in the liver, and the content ratio based on the number of molecules was calculated, including both the ester (mono-, di-, and triglycerides) and free forms. The numbers of –CH and –CH_2_, which exhibit typical spectra in the NIR region, were calculated for each fatty acid. Because CH is present in the hydrocarbon chain with double bonds (hydrogen unsaturation), the fraction of CH_2_ groups from the sum of the CH and CH_2_ numbers represents the DS of the hydrocarbon chain. Therefore, the ratio [CH_2_/(CH + CH_2_)] was calculated for each sample and identified as the DS. Similarly, [{CH_3_ + CH_2_ + CH + 1(COOH)}/CH_3_] was identified as HCL, where –CH_3_ only existed at the terminus of the hydrocarbon chain. For example, the HCL of palmitic acid (C16:0) and stearic acid (C18:0) are 16 and 18, respectively, while their DS are 1. The HCL of oleic acid (C18:1), LA (C18:2), and α-linolenic acid (C18:3) are 18, while their DS values are 0.88, 0.75, and 0.63, respectively. Table 1 shows the average values of HCL and DS of fatty acids in each liver lobe determined by GC for each group of mice.

**Table 1:** Lipid contents of liver tissues of mice fed with each diet analyzed by GC and the Folch method.

Diet (number of liver lobes)	Total lipid content (mg/g)	HCL of fatty acids	DS of fatty acids
ND (*n* = 18)	66.2 ± 20.2 (46.0–86.4)	17.72 ± 0.07 (17.64–17.79)	0.77 ± 0.01 (0.76–0.78)
HFD (*n* = 31)	135.0 ± 54.2 (80.8–189.2)	17.56 ± 0.72 (17.49–17.63)	0.79 ± 0.01 (0.78–0.80)
HCD (*n* = 34)	174.2 ± 35.8 (138.3–210.0)	17.83 ± 0.23 (17.81–17.85)	0.75 ± 0.01 (0.74–0.76)
HFD (2% LA) (*n* = 12)	80.4 ± 27.8 (52.6–108.3)	17.22 ± 0.11 (17.11–17.34)	0.83 ± 0.01 (0.82–0.84)
HFD (12% LA) (*n* = 14)	65.2 ± 24.2 (41.0–89.4)	17.54 ± 0.08 (17.45–17.62)	0.81 ± 0.01 (0.80–0.82)

Data are presented as mean ± standard deviation (SD). The minimum and maximum HCL and DS of fatty acids in the livers of mice fed with each diet are also shown.

*HCD* high-cholesterol diet, *HFD* high-fat diet, *LA* linoleic acid [18:2 (n-6)], *ND* normal diet.

**Figure 2:**
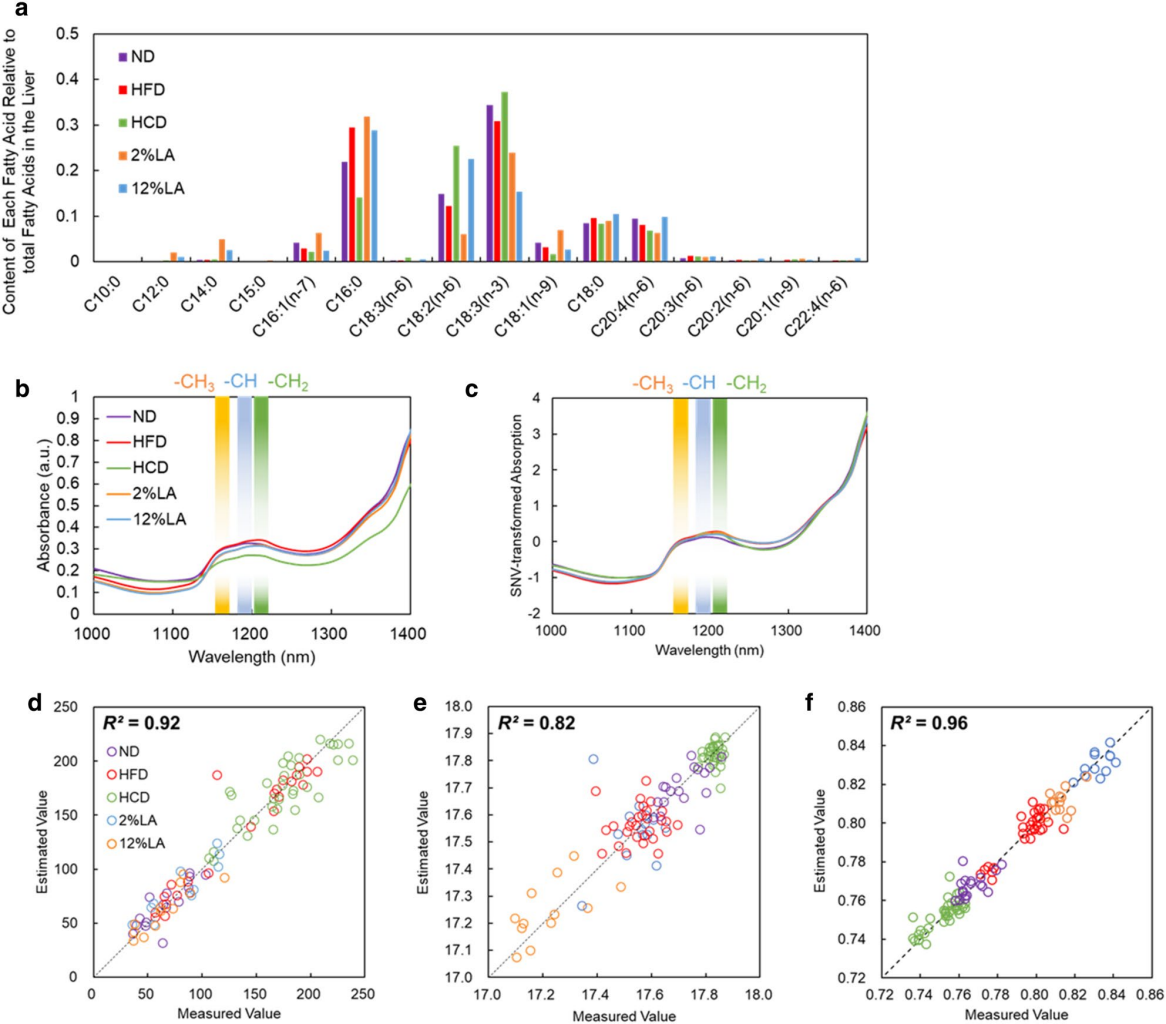
NIR spectra provided for analysis of lipid content in the liver with information on the chemical properties of fatty acids. (**a**) Contents of the 16 fatty acids, determined by GC, in the liver of mice fed with each diet. (**b,c**) Averaged absorption spectra (**b**) without and (**c**) with SNV correction for liver tissues of mice fed with different chows (12% LA [18:2 (n-6)], 2% LA, HCD, HFD, and ND). (**d–f**) Results of SVR analysis on the NIR spectra with SNV to visualize the distribution of (**d**) total lipid content, (**e**) HCL, and (**f**) the DS of fatty acids in the livers of mice fed with each diet.

The average HCL of fatty acids in each liver lobe sample ranged from 17.11 to 17.85, with the values increasing in the order of 2% LA, 12% LA, HFD, ND, and HCD. The livers of mice fed with any of the diets had high contents of three fatty acids, palmitic acid (C16:0), LA (C18:2), and α-linolenic acid (C18:3) (Fig. 2a), and the average HCL ranged from 16 to 18. The average DS was in the range of 0.74–0.84, with the values increasing in the order of HCD, ND, HFD, 12% LA, and 2% LA. This indicates that the percentage of double bonds in the hydrocarbon chain of fatty acids decreased in the liver in this order.

### NIR absorption spectra of fatty acids and liver tissues

The NIR absorption spectra of the liver samples were observed in the hyperspectral images of samples collected from mice fed with different diets. The potential effect of baseline drift correction by the standard normal variate (SNV) was also examined. Figure 2b depicts the average absorption spectra of 1000 pixels in each group from the NIR-HSI data of the livers of mice fed with each diet. In the absorption spectra after SNV processing (Fig. 2c), focusing on the wavelength range of 1150–1210 nm, there was a difference in the shape of the absorption peak for each diet. Using the SNV-transformed spectra, the accuracy of SVR in estimating the total lipid content (Fig. 2d), HCL (Fig. 2e), and DS (Fig. 2f) was determined for each liver lobe. For each lobe, the estimated values obtained by SNR analysis of the NIR-HSI data were plotted against the measured values determined by the Folch^16^ method and GC^18^. Notably, HCL (Fig. 2e) and DS (Fig. 2f) clearly showed the characteristic clustering of the plotted dots for each diet. In addition to the total lipid content (Fig. 2d) reported in our previous study^13^, the DS (Fig. 2f) was also estimated with a high coefficient of determination (*R*^2^ > 0.9) by SVR analysis of the NIR-HSI data. HCL (Fig. 2e), calculated using the number of –CH_3_ groups, was also estimated from the NIR-HSI data with high accuracy (*R*^2^ = 0.82). As only one –CH_3_ group exists in a linear fatty acid molecule, the absorption derived from CH_3_ may not be high. In addition, the absorption of other functional groups also appears near the absorption peak of –CH_3_ (at approximately 1150 nm), close to the absorption of the second harmonic of CH_2_ at 1170–1180 nm^19,20^. The relatively low accuracy of HCL estimation may be attributed to the presence of various spectra of such non-target functional groups; therefore, further investigation is needed to improve accuracy.

### Two-dimensional mapping and visualizing the distribution of HCL and DS of fatty acids in liver tissue

**Figure 3:**
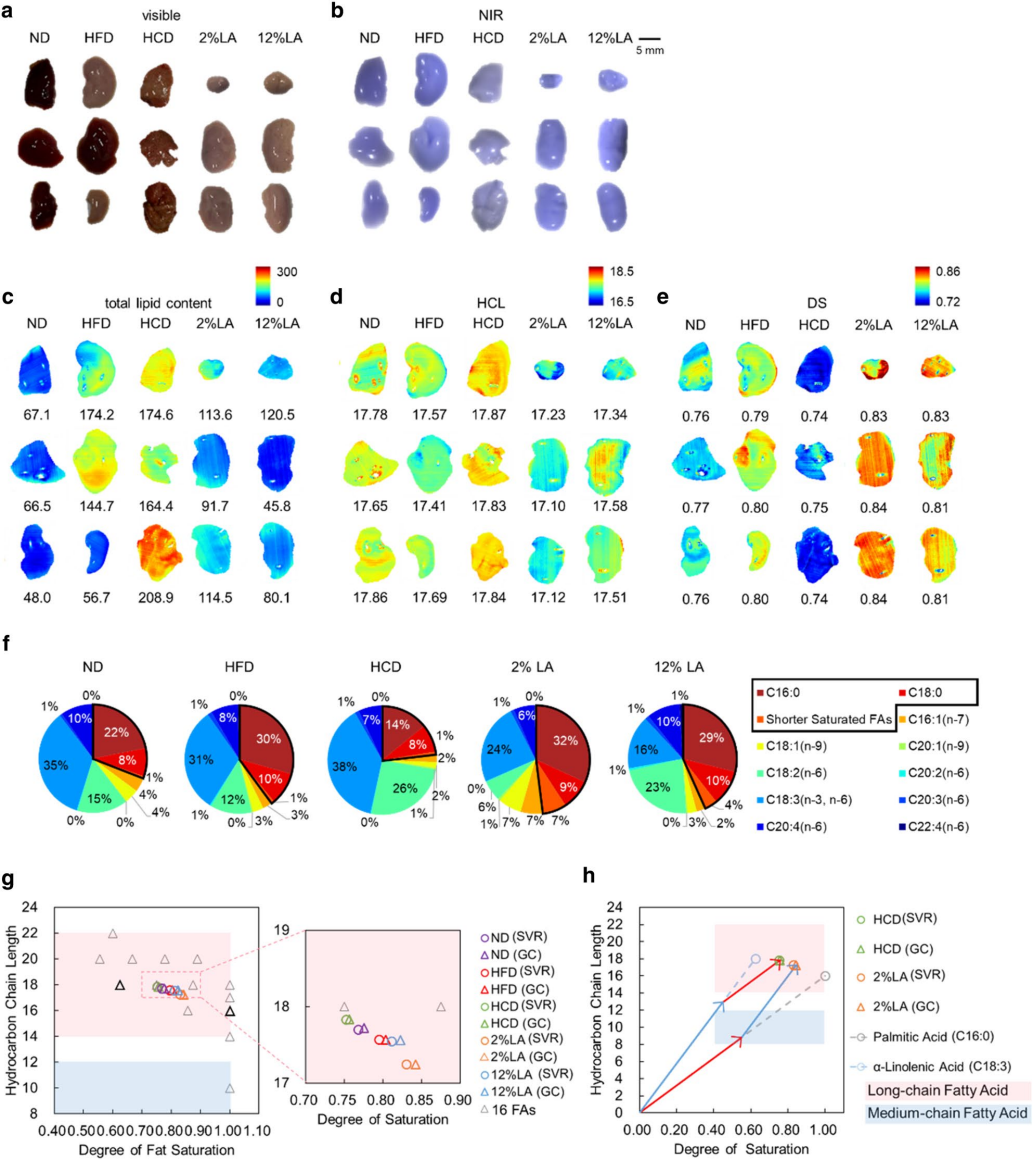
NIR-HSI to visualize the distribution of the HCL and DS of fatty acids in the liver. (**a**) Visible and (**b**) pseudo-color NIR images (R: 1210 nm, G: 1190 nm, B: 1080 nm) of hepatic lobes of mice fed with each diet. (**c–e**) Result of SVR analysis of NIR hyperspectral images to visualize the distribution of (**c**) total lipid content, (**d**) HCL, and (**e**) DS in the hepatic lobes of mice. (**f**) Pie charts of contents of the 16 fatty acids, determined by GC, in the liver of mice fed with each diet. (**g,h**) Scatter plots of the chemical components of fatty acids in the lipid and total lipid contents of the liver demonstrated by NIR-HSI. (**g**) Plots of averaged vectors of the fatty acid information contained in the liver of mice fed with each diet. The vectors of each fatty acid are also shown. An enlarged view on the right shows the varied position of the dots on the HCL/DS plot, indicating that the proportion of the accumulated fatty acids varies with the diets fed. (**h**) A model of combined vectors that indicate fatty acid composition in tissue samples. The red and blue vectors represent palmitic acid and α-linolenic acid, respectively.

Next, the NIR absorption spectra at each pixel obtained by NIR-HSI of the liver lobes (Fig. 3a,b) were subjected to SVR analysis to visualize the in situ distribution of the total lipid content (Fig. 3c) as well as the HCL (Fig. 3d) and DS (Fig. 3e) of the fatty acids. As shown in the mapping results (Fig. 3c–e) and the fatty acid content of each group (Figs. 2a, 3f), the livers of mice fed with HFD had high DS owing to the high palmitic acid (C16:0) content, whereas the HCD group had low DS owing to the high LA (C18:2) and α-linolenic acid (C18:3) content. The livers of mice fed with a diet with 2% LA (low LA), an unsaturated fatty acid, had a smaller HCL and a higher DS because they contained palmitic acid and the shorter myristic acid (C14:0), which are saturated fatty acids, at high concentrations. Mice fed with a 12% LA diet with more LA also had higher LA content in the liver. However, as the liver of the 12% LA diet group contained low levels of the trivalent unsaturated fatty acid α-linoleic acid, they exhibited slightly higher DS values.

Then, the characteristic fatty acid composition that occurs in the livers of mice fed with each diet is depicted in the two-dimensional plot of HCL and DS (Fig. 3g,h). The dots representing the livers in the HCL/DS plot can be recognized as linear combinations of the vectors of fatty acid content (Fig. 3h). Owing to the high content of palmitic acid, LA, and α-linoleic acid in the livers of all groups (Fig. 2a), the plot, expressed as average HCL/DS in the tissue, considers values within the range connected by the dots of these three major fatty acids. Even within this range, the position of the dots that appeared on the HCL/DS plot changed according to the diet-induced alterations in the content ratios of accumulated fatty acids (Fig. 3g).

### Visualizing the distribution of HCL and DS of fatty acids in tumor-bearing liver tissue

**Figure 4:**
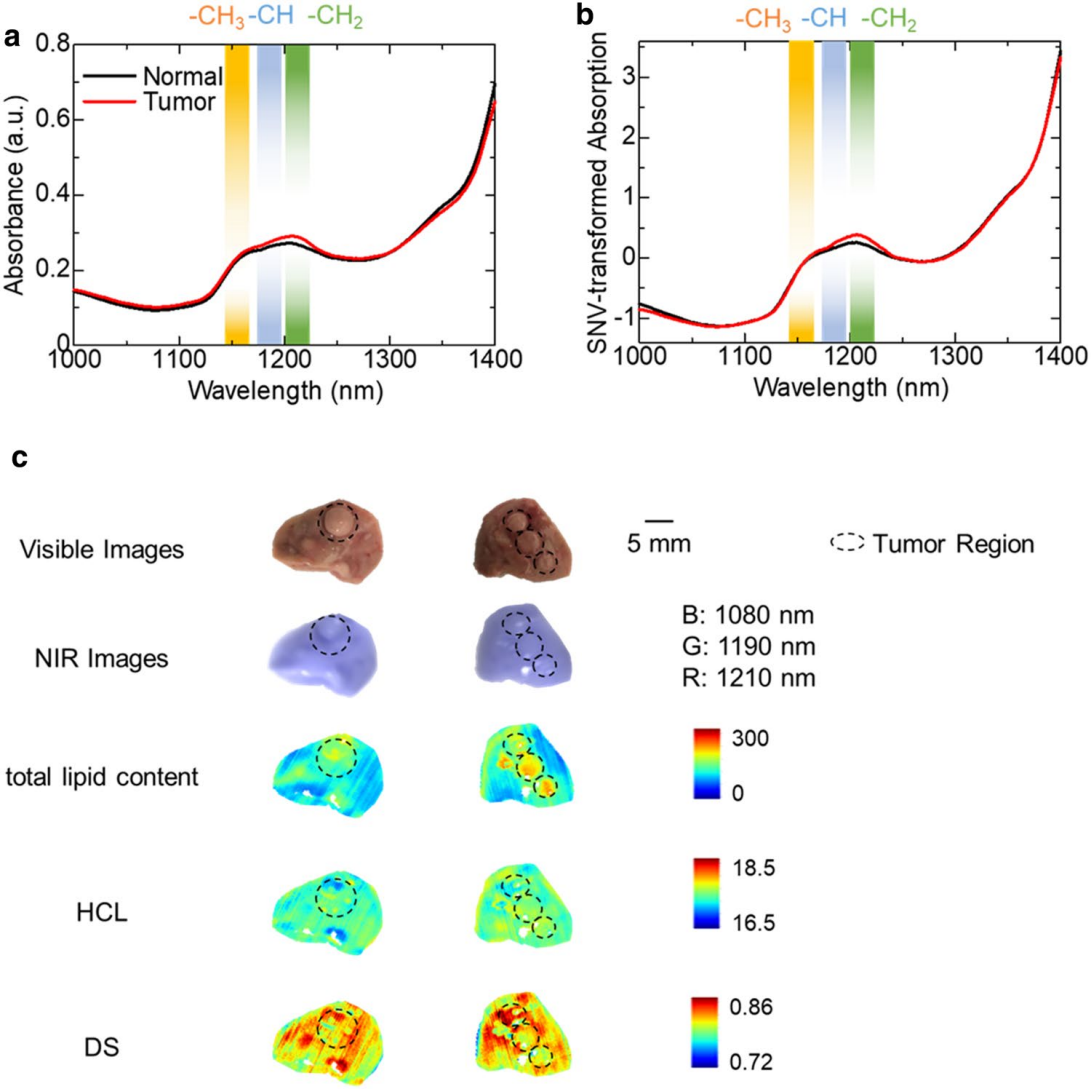
NIR-HSI visualizes the distribution of HCL and fatty acid DS in the tumor-bearing liver. (**a,b**) Averaged absorption spectra (**a**) without and (**b**) with SNV correction for tumor-bearing liver tissues of mice. (**c**) Visible and pseudo-color NIR images (R: 1210 nm, G: 1190 nm, B: 1080 nm) of the hepatic lobes and results of SVR analysis of NIR hyperspectral images to visualize the distribution of total lipid content, HCL, and DS in the hepatic lobes of mice.

Finally, the NIR absorption spectra of liver samples were observed in hyperspectral images of samples taken from tumor-bearing mice fed with HFD containing 12% LA. The potential effect of baseline drift correction by the SNV was also examined. Figure 4a represents the average absorption spectra of 100 pixels from the NIR-HSI data in normal and tumor areas of the liver. In the absorption spectrum after SNV processing, there was a difference in the shape of the absorption peak in the wavelength range of 1150–1210 nm between the normal and tumor areas (Fig. 4b). In particular, a shape difference appeared in the 1185–1195 nm wavelength region, where the absorption peak of the second harmonic of –CH appears, suggesting that the types of fatty acids accumulated in the tumor and normal areas were different.

The NIR absorption spectra at each pixel obtained by NIR-HSI of the liver lobes were analyzed by SVR to visualize the distribution of the total lipid content, and HCL and DS of the fatty acids (Fig. 4c). Compare to the average total lipid content of 123.3 mg/g in the whole liver lobes, it was higher in tumor areas (150.0 mg/g). The NIR-HSI data also showed that, while there was no difference in the HCL between the tumor and normal areas, the DS was lower in the tumor compared to the normal area (Fig. 4c); probably because the fatty acids accumulated in the tumors contained more dietary unsaturated fatty acids (high LA content). As demonstrated by the results, the methods of NIR-HSI analysis has a potential to visualize the contrast of fatty acid contents in lesion tissues such as liver cancer. The results of cancer in mice fed with ND and the potential relationship between lipid accumulation profile and carcinogenesis are the subjects that should be investigated in future studies.

## Discussion

Label-free imaging facilitates the observation of small animals and organ tissues while maintaining the integrity of samples. Here, we introduced a nonlinear regression model for NIR-HSI analysis using the content of 16 major fatty acids (Suppl. Table S2) in the liver as training data, enabling new capabilities to image the distribution of characteristic fatty acids in situ. As shown in our previous study^13^, the estimation error in such NIR-HSI is minimized (relative error < 10%) by preparing training data using more than 70 samples. Therefore, the present study, which used more than 100 samples with variations in fatty acid composition, can be considered to have been conducted with sufficient supervised data. Our findings from the two-dimensional mapping of the HCL and DS of fatty acids in the liver revealed characteristic clusters of fatty acids in the liver according to the different ingested diets. Although we focused mainly on the lipid bands shown in Figs. 2c and 4, the optical absorption of biological molecules in the NIR region is complex, as evidenced by the validity of the nonlinear SVR model. There may be further absorption bands for investigating liver disease and metabolism. The non-destructive analysis of these fatty acids can be a valuable tool for elucidating the pathogenesis of diseases involving lipid metabolism^21^ and the physiological roles of unsaturated fatty acids^22,23^. Although the NIR-HSI method with machine learning presented here has thus been applied only to ex vivo organs, the most significant advantage of this method is its potential for mapping the distribution of fatty acids with their characteristic chemical structures without slicing, homogenizing, or chemically staining organ samples (Fig. 3). HSI techniques reportedly have the potential for food quality screening^11^ and detecting submucosal tumors^12^. In this study, we demonstrated that the distribution of molecules related to metabolism and pathophysiology in the liver could also be visualized in situ based on the chemical structure using NIR-HSI and machine learning with well-prepared training data. This label-free imaging technique, together with the establishment of further imaging systems, can potentially be used for live imaging in the biomedical field in the future, e.g., an NIR-HSI camera equipped with a laparoscope^24^. NIR-HSI data contains huge and complex information that requires machine learning-based analysis techniques such as SVR. The advantage of HSI is that it can obtain information from a large amount of light without using specific optical filters such as those used in Raman spectroscopy. Ultrasonography^25,26^, computed tomography^27^, and magnetic resonance imaging^27,28^ have been used as techniques for detecting liver diseases such as NAFLD and cancer. Compared to such existing techniques, NIR-HSI is shown to uniquely visualize the distribution of molecular information of fat in biological tissues. The newly presented parameters in this study, HCL and DS, are factors that determine the properties of fat molecules and their biological effects. It would be interesting to elucidate the relationship between the parameters and pathogenesis of diseases such as NAFLD. Furthermore, NIR-HSI will be an effective imaging analysis tool not only for clinical applications, but also for biological and physiological research, as it enables mapping of molecular information.

Overall, in this study, 16 fatty acids were selected as common lipid components in the mouse liver and categorized based on HCL and DS. On each lobe of the mouse liver, by analyzing NIR (1000–1400 nm) spectra acquired using hyperspectral imaging using machine learning, SVR, the average values of the HCL and DS of fatty acids were well predicted by this method for each lobe in addition to the total lipid concentration. The HCL and DS concentration distributions of various types of fatty acids in terms of these two parameters were graphically depicted based on the analysis. The analysis also demonstrated that in the two-dimensional plots of HCL and DS, characteristic accumulation of the depicted dots of fatty acids in the livers of mice fed with different diets was observed. Visualization of lipid distribution in higher-dimensional information rather than simply using total lipid content as a single parameter would provide a novel tool for revealing the pathophysiological conditions of liver diseases and metabolism.

## Methods

### Materials

Chloroform, methanol, acetyl chloride, and hexane were purchased from Fujifilm Wako Pure Chemical Co. (Osaka, Japan). Heptadecanoic acid (C17:0) was purchased from Sigma-Aldrich (St. Louis, MO, USA). Hydrochloric acid (2%) in methanol (2% HCl/MeOH) was prepared by mixing acetyl chloride (5 mL) and methanol (35 mL) on ice.

### Animals and collection of liver samples

All protocols using mice were carried out with minimizing the number of mice used and their suffering. In accordance with national and institutional guidelines under approval by the Animal Care and Use Committees of Tokyo University of Science (approval number: K22004) and Osaka Metropolitan University (approval number: 2021-088). All methods are reported in accordance with ARRIVE guidelines. C57BL/6NJcl and BALB/c mice were purchased from CLEA Japan, Inc. (Tokyo, Japan) and Japan SLC, Inc. (Shizuoka, Japan), respectively. The mice were housed under pathogen-free conditions with a 12-h/12-h light/dark cycle. They were fed with either an ND (AIN-76A; Research Diets, Inc., NJ, USA), high-fat diet (HFD) (D12492; 60% fat by energy with lard-rich; Research Diets, Inc.), high-cholesterol diet (HCD) (D12336; 35% fat by energy with cocoa butter, coconut oil, and additive 1.5% cholesterol; Research Diets), or modified HFD with 2% LA (D17011206; Research Diets, Inc.) or 12% LA (D17011207; Research Diets Inc.) for 5 days to 32 weeks (Suppl. Table S1). The HFDs with 2% and 12% LA, designed as diets for investigation of hepatic carcinogenesis via NAFLD^29^, were prepared by replacing lard and soybean oil with defined ratios of coconut oil (rich in saturated fatty acids with low LA) and safflower oil (high LA). Such a variety of diet feeding rendered mice harboring a wide range of lipid components in the liver with various characteristics, such as HCL and DS. Liver samples were isolated from the mice under anesthesia using 2.0% isoflurane and subjected to NIR-HSI analysis. NIR hyperspectral images (wavelength: 760–1885 nm; band interval: 2.2 nm) were taken for each liver lobe.

### Chemically induced liver carcinogenesis

Male mice were treated with 7,12-dimethylbenz(*a*)anthracene (DMBA) (Sigma) for carcinogenesis experiments as described previously^30^. In brief, 50 μL of 0.5% DMBA in acetone was applied to the dorsal surface on postnatal day 4–5. The neonatal mice used for carcinogenesis experiments were shuffled from their littermates when grouping to avoid any littermate tendency. Afterwards, mother and pups were fed with HFD. Pups were weaned at 4 weeks old and continuously fed with HFD containing 12% LA until euthanized at 34 weeks old.

### Lipid extraction and weighing total lipid

Liver samples were weighed using an electronic scale and subjected to NIR-HSI before lipid extraction. Total lipids were extracted from each sample using the Folch method^16^, an established protocol for isolating lipids from animal tissues. Briefly, the sample was minced using a BioMasher (Nippi, Inc., Tokyo, Japan) in 7 mL of 2:1 (v/v) chloroform–methanol mixture. After adding 2 mL distilled water to the lipid-containing mixture, it was centrifuged at 1400×*g* for 7 min. The organic fraction was then placed in a glass tube and heated to 60 °C until the organic solvent completely evaporated. The lipids remaining in the glass tubes was weighed using an electronic scale.

### GC analysis

The fatty acid content was analyzed for the lipids extracted from each liver lobe sample. The concentration of each fatty acid was analyzed by GC for the extracted lipid samples of each liver lobe collected from ND-fed and lipid-controlled-fed mice to acquire representative information on a series of fatty acids^18^. After lipid extraction, heptadecanoic acid (1 mg) was added to the reaction mixture as the internal standard. The extracted lipids were dissolved in 2% HCl/MeOH (2 mL) at 55 °C for 20 min for methylation. Subsequently, distilled water (1 mL) and *n*-hexane (3 mL) were added to the fatty acid methyl esters and vortexed. The samples were centrifuged at 3000×*g* for 10 min, and the n-hexane fraction was concentrated by evaporation under reduced pressure. The fatty acid methyl esters were extracted with chloroform and analyzed by gas–liquid chromatography using a gas chromatograph (Nexis GC-2030; Shimadzu Co., Kyoto, Japan) equipped with a flame ionization detector (FID) and a split-injection system (1:50 split ratio) fitted with a capillary column (Supelco SPB-1; 30 m length × 0.25 mm i.d.; Sigma-Aldrich). The initial column temperature was 180 °C for 30 min and subsequently increased to 210 °C at a rate of 60 °C/min. The column temperature was maintained at 210 °C for 29.5 min. The injector and detector were operated at 250 °C. Helium was used as the carrier gas at a flow rate of 1.4 mL/min. The fatty acid peaks were identified by comparing the retention times with those of known standards (Suppl. Fig. S1). The amount of each fatty acid was determined using 1 mg of the internal and external standards.

### NIR-HSI and data analysis by SVR

A line-scanning NIR-HSI system was used to obtain hyperspectral images of the samples, as shown schematically in Fig. 1. The system comprises an NIR reflectance imaging spectrometer (ImSpector N17E, Specim, Oulu, Finland) coupled with an NIR camera (XEVA-1.7-640, Xenics nv, Leuven, Belgium) and NIR objective lens (SWIR series 83–160, focal length 25 mm, Edmund Optics, NJ, USA), a gold-coated rotation mirror (40 × 100-mm^2^) for scanning the y-axis, halogen lamp (LA-150UE, Hayashi-Repic Co., Ltd., Tokyo, Japan) as a light source, and computer (JFE Techno-Research Co., Tokyo, Japan)^13^ (Fig. 1b). The NIR camera includes a two-dimensional indium gallium arsenide (InGaAs) sensor array that acquires images of 512 × 640 pixels corresponding to the wavelength and position of NIR light (900–1700 nm; wavelength resolution: 2.2 nm) in this coupled system. In a single shot using the NIR-HSI system, the optical intensity values for each wavelength band at each pixel on a line are captured. The y-axis line scan reconstructs intensity (reflectance ratio) maps for each wavelength on the x–y pixel array. The power of the halogen lamp was controlled so that the sample temperature was stable. HSI data acquisition was performed for liver samples in a dark room at a temperature and humidity of approximately 22–24 °C and 50–60%, respectively. The acquisition was performed after recording dark and white references to obtain reflectance ratio for each wavelength band, then the data were preprocessed as previously described^13^. The sizes of the region of interest were 1500–3000 pixels per a sample. The NIR reflectance images were normalized to determine the relative reflectance *R*(*λ*) using the following equation:

1 $$\begin{array}{c}R\left(\lambda \right)=\frac{{I}_{raw}\left(\lambda \right)-{I}_{dark}\left(\lambda \right)}{{I}_{white}\left(\lambda \right)-{I}_{dark}\left(\lambda \right)}\end{array},$$

where *R*(λ) is the calculated relative reflectance for each λ, *I*_*raw*_(λ) is the raw intensity of a given pixel, *I*_*dark*_(λ) is the dark reference image for converting the intensity images into reflectance images by removing the effect of the dark current, and *I*_*white*_(λ) is the intensity obtained from the white reference image. After removing spikes in the NIR images, all pixels belonging to each liver sample were selected. Furthermore, the mean reflectance spectrum for each liver sample was calculated from the component pixel reflectance spectra and used to represent the liver samples. In addition, the absorbance spectra corresponding to these reflectance spectra, defined as log(1/*R*(*λ*)), were used for the analysis. Spectra of wavelength ranges < 1000 nm and > 1400 nm were removed from the analysis because of the lower sensitivity of the NIR camera and high absorption by water in those ranges.

The reflectance spectral data at pixels were analyzed by SVR, a nonparametric supervised machine learning algorithm^31^ that can transform a nonlinear regression problem into linear regression by implementing a kernel function^32^. Since SVR has been shown as the suitable regression model for NIR-HSI data analysis by comparing results with those obtained using partial least squares regression (PLSR)^13^, SVR was also used in the present study. To compensate for the influence of scattering on the sample surface and the dark-current noise of the camera, the NIR reflectance spectra were normalized by SNV using the mean and standard deviation of the spectrum of interest^33^ as follows:

2 $$\begin{array}{c}z =\frac{x-\mathrm{mean}\left(x\right)}{\mathrm{std}\left(x\right)},\end{array}$$

where *x* and *z* are the original and SNV-transformed spectra, respectively, and mean(*x*) and std(*x*) are the mean and standard deviation of *x* over the entire wavelength range. Internal validation was performed using the leave-one-out cross-validation method to generate the SVR calibration models. The SNV is also able to normalize the data for each set of experiment in order to compare the whole data set. Training data were generated by matching the NIR spectral data with the average values of the total lipid content and the HCL and DS of fatty acids determined using the Folch extraction method and GC. The models were evaluated using the *R*^2^ of cross-validation, defined as follows:

3 $$ \begin{array}{*{20}c} {R^{2} = 1 - \frac{{\mathop \sum \nolimits_{i}^{n} \left( {\hat{y}_{i} - y_{i} } \right)^{2} }}{{\mathop \sum \nolimits_{i}^{n} \left( {\hat{y}_{i} - \overline{{y_{i} }} } \right)^{2} }}} \\ \end{array} , $$

where $\hat{y}_{i}$ and *y*_*i*_ are the measured and predicted values for the *i*-th liver sample, respectively, n is the number of samples, and $\overline{y}$ is the average of the measured values of all samples.

### HCL/DS plotting

To visualize the changes in fatty acid content in the liver owing to different diets, the mean values (for each diet group) of HCL and DS of fatty acids in the liver estimated by NIR-HSI and validated by GC were plotted on a two-dimensional plane with HCL and DS represented on the x and y axes, respectively.

### Analysis of tumor-bearing mice by SVR

Livers were harvested under anesthesia from tumor-bearing mice and performed the NIR-HSI analysis. NIR hyperspectral images were obtained for each liver lobe. The reflectance spectral data of the tumor-bearing mice were analyzed by SVR using the data set of 102 samples shown in Suppl. Table S1 to visualize the distribution of total lipid content, HCL and DS.

### Supplementary Information

Supplementary Information.

## Data Availability

The datasets used and analyzed during the current study are available from the corresponding author on reasonable request.
