# Supplementary material for: Visualization of hydrocarbon chain length and degree of saturation of fatty acids in mouse livers by combining near-infrared hyperspectral imaging and machine learning
Source: Sci Rep. 2023 Nov 23;13:20555. doi: 10.1038/s41598-023-47565-z (PMC10667523; doi:10.1038/s41598-023-47565-z)
Supplement: Supplementary file 1 — Supplementary Information. [file 41598_2023_47565_MOESM1_ESM.docx]

# Supplementary Information

# Visualization of Hydrocarbon Chain Length and Degree of Saturation of Fatty Acids in Mouse Livers by Combining Near-Infrared Hyperspectral Imaging and Machine Learning

Akino Mori^1^, Masakazu Umezawa^1,^*, Kyohei Okubo^1^, Tomonori Kamiya^2^, Masao Kamimura^1^, Naoko Otani^2^, Kohei Soga^1,^*

^1^ Department of Materials Science and Technology, Faculty of Advanced Engineering, Tokyo University of Science, 6-3-1 Niijuku, Katsushika, Tokyo 125-8585, Japan

^2^ Department of Pathophysiology, Osaka Metropolitan University, Graduate School of Medicine, 1-4-3 Asahimachi, Abeno, Osaka 545-8585, Japan

*Corresponding authors

**Suppl. Table S1. Number of samples analyzed in this study**

| Feed | Number of mice | Number of hepatic lobes analyzed |
| --- | --- | --- |
| ND | 10 | 18 |
| HFD | 9 | 23 |
| HCD | 9 | 35 |
| HFD (2% LA) | 6 | 12 |
| HFD (12% LA) | 7 | 14 |
| Total | 41 | 102 |

Abbreviations: HCD, high-cholesterol diet; HFD, high-fat diet; LA [18:2 (n-6)], linoleic acid; ND, normal diet.

**Suppl. Table S2. Sixteen fatty acids analyzed by GC**

| Fatty acid name | Abbreviation | Structural formula |
| --- | --- | --- |
| Capric Acid | C10:0 |  |
| Lauric Acid | C12:0 |  |
| Myristic Acid | C14:0 |  |
| Pentadecanoic acid | C15:0 |  |
| Palmitoleic Acid | C16:1 (n-7) |  |
| Palmitic Acid | C16:0 |  |
| γ-Linolenic Acid | C18:3 (n-6) |  |
| Linoleic Acid | C18:2 (n-6) |  |
| α-Linolenic Acid | C18:3 (n-3) |  |
| Oleic Acid | C18:1 (n-9) |  |
| Stearic Acid | C18:0 |  |
| Arachidonic Acid | C20:4 (n-6) |  |
| Dihomo-γ-Linolenic Acid | C20:3 (n-6) |  |
| 11(Z), 14(Z)-Eicosadienoic acid | C20:2 (n-6) |  |
| 11(Z)-Eicosenoic acid | C20:1 (n-9) |  |
| Adrenic acid | C22:4 (n-6) |  |


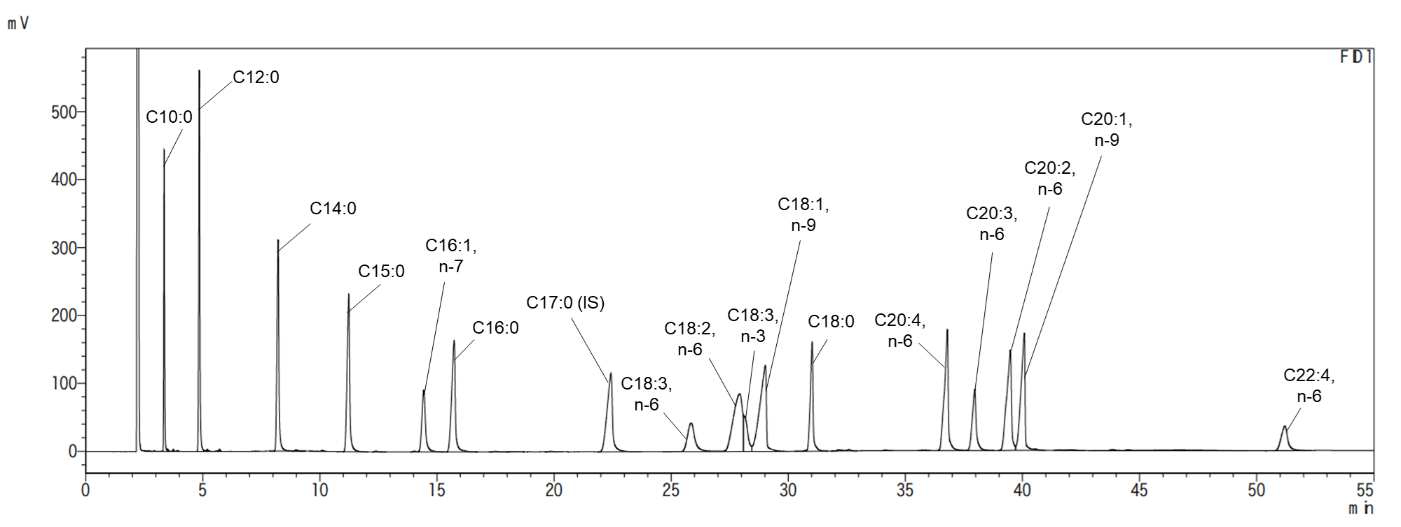
**Suppl. Fig S1. Representative chromatogram of fatty acid in GC-FID.** C10:0, capric acid; C12:0, lauric acid; C14:0, myristic acid; C15:0, pentadecanoic acid; C16:1,n-7, palmitoleic acid; C16:0, palmitic acid; C17:0, heptadecanoic acid (internal standard); C18:3,n-6, γ-linolenic acid; C18:2,n-6, linoleic acid; C18:3,n-3, α-linolenic acid; C18:1,n-9, oleic acid; C18:0, stearic acid; C20:4,n-6, arachidonic acid; C20:3,n-6, dihomo-γ-linolenic acid; C20:2,n-6, 11(Z), 14(Z)-eicosadienoic acid; C20:1,n-9, 11(Z)-eicosenoic acid; C22:4,n-6, adrenic acid.
